# Ancient Origin of the Modern Deep-Sea Fauna

**DOI:** 10.1371/journal.pone.0046913

**Published:** 2012-10-10

**Authors:** Ben Thuy, Andy S. Gale, Andreas Kroh, Michal Kucera, Lea D. Numberger-Thuy, Mike Reich, Sabine Stöhr

**Affiliations:** 1 Geoscience Centre, University of Göttingen, Department of Geobiology, Göttingen, Germany; 2 School of Earth and Environmental Sciences, University of Portsmouth, Portsmouth, United Kingdom; 3 Natural History Museum Vienna, Department of Geology and Palaeontology, Vienna, Austria; 4 Marum – Centre for Marine Environmental Sciences, University of Bremen, Bremen, Germany; 5 Geoscience Centre, Museum, Collections and Geopark, University of Göttingen, Göttingen, Germany; 6 Swedish Museum of Natural History, Stockholm, Sweden; Ludwig-Maximilians-Universität München, Germany

## Abstract

The origin and possible antiquity of the spectacularly diverse modern deep-sea fauna has been debated since the beginning of deep-sea research in the mid-nineteenth century. Recent hypotheses, based on biogeographic patterns and molecular clock estimates, support a latest Mesozoic or early Cenozoic date for the origin of key groups of the present deep-sea fauna (echinoids, octopods). This relatively young age is consistent with hypotheses that argue for extensive extinction during Jurassic and Cretaceous Oceanic Anoxic Events (OAEs) and the mid-Cenozoic cooling of deep-water masses, implying repeated re-colonization by immigration of taxa from shallow-water habitats. Here we report on a well-preserved echinoderm assemblage from deep-sea (1000–1500 m paleodepth) sediments of the NE-Atlantic of Early Cretaceous age (114 Ma). The assemblage is strikingly similar to that of extant bathyal echinoderm communities in composition, including families and genera found exclusively in modern deep-sea habitats. A number of taxa found in the assemblage have no fossil record at shelf depths postdating the assemblage, which precludes the possibility of deep-sea recolonization from shallow habitats following episodic extinction at least for those groups. Our discovery provides the first key fossil evidence that a significant part of the modern deep-sea fauna is considerably older than previously assumed. As a consequence, most major paleoceanographic events had far less impact on the diversity of deep-sea faunas than has been implied. It also suggests that deep-sea biota are more resilient to extinction events than shallow-water forms, and that the unusual deep-sea environment, indeed, provides evolutionary stability which is very rarely punctuated on macroevolutionary time scales.

## Introduction

The deep sea is by far the largest environment on the planet, yet our knowledge of the diversity and evolutionary history of the deep-sea biota remains remarkably poor [1]. Early deep-sea research in the nineteenth century led to the discovery of peculiar organisms like stalked crinoids (sea lilies), until then known only as fossils from shallow-marine deposits. Such finds were the origin of the idea that the deep sea is a refuge for ancient lineages excluded from shelf habitats by superior competitors or increased predation pressure [2].

Although the antiquity of at least part of the deep-sea fauna is implicit in the notion of the apparent offshore migration of marine benthos over time [3], debates have increasingly focused on the impact of Mesozoic dysoxia (Oceanic Anoxic Events: OAEs) and the Cenozoic cooling of deep-water masses on the colonization of deep habitats [1], [4], [5]. Biogeographic patterns and molecular clock estimates calibrated against the fossil record of shallow-water sister groups of the modern deep-sea fauna have yielded dates from early Mesozoic to Pleistocene, with the majority converging to a latest Mesozoic or Cenozoic origin [5], [6], [7], [8].

**Figure 1:**
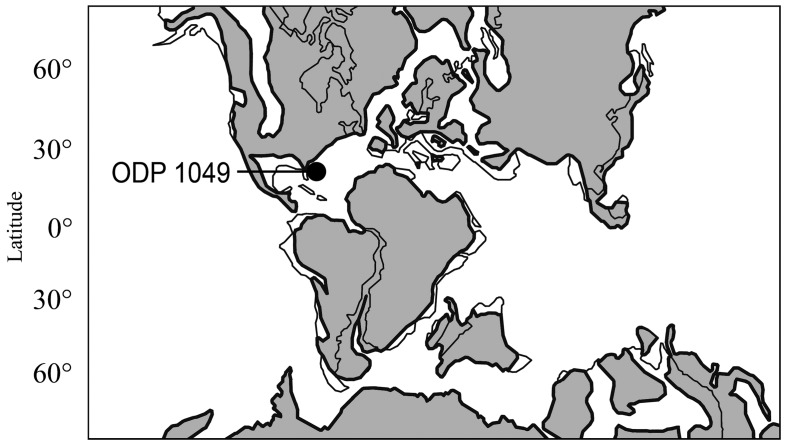
Paleogeographic reconstruction for the late Aptian with the position of ODP site 1049 (Blake Nose). Thick lines denote paleo-coastlines, grey areas represent emerged land (modified from ref. [51]).

The reason why this debate is unsettled is the lack of direct evidence of the geological history of deep-sea fauna due to the absence of outcrops of deep-sea sediments with preserved fossil remains [6]. While oceanic sediment cores provide an excellent fossil record of benthic foraminifers and ostracods, they have been assumed to almost never yield identifiable remains of larger deep-sea benthos [6].

Modern communities of deep-sea megabenthos are commonly dominated by echinoderms, both in terms of abundance and biomass [9]. In particular, ophiuroids (brittle stars) and holothuroids (sea cucumbers) can cover the deep-sea floor in dense concentrations of hundreds to thousands of individuals per square meter [10]. The echinoderm skeleton is made up of numerous plates composed of high Mg-calcite that have a high fossilization potential. Although the skeletal elements usually dissociate after death, many are morphologically diagnostic to genus or even species level, e.g. [11]–[12]. Therefore, echinoderms are likely to have left a fossil record in deep-sea deposits, and thus can provide insights into the composition and diversity of ancient deep-sea faunas.

Here, we describe an Early Cretaceous echinoderm assemblage providing the first fossil-based insights into Mesozoic deep-sea biodiversity. We compare the composition of the echinoderm assemblage with that of present-day equivalents and coeval shallow-water assemblages and discuss the implications for the origin of the modern deep-sea fauna.

**Figure 2:**
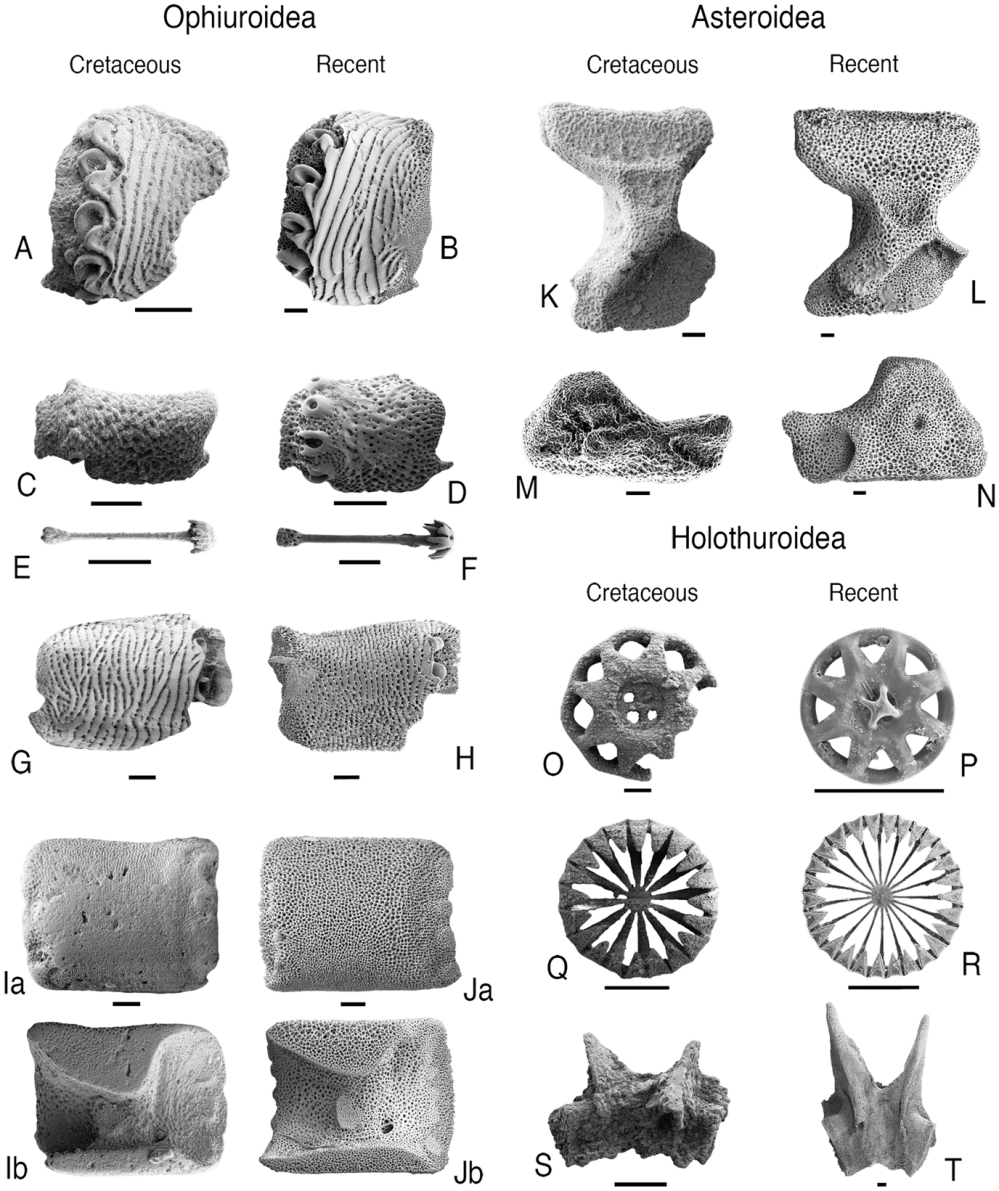
Key echinoderm plates from the early Cretaceous of Blake Nose (ODP site 1049) with corresponding plates of Recent relatives. A: *Ophiolimna* sp. (Ophiacanthidae), lateral arm plate (LAP), Blake Nose (GZG.INV.78777). B: *Ophiolimna bairdi* (Lyman) (Ophiacanthidae), LAP, Recent, North Atlantic. C: Ophiohelinae gen. nov. (Ophiacanthidae), LAP (GZG.INV.78778), Blake Nose. D: *Ophiotholia spathifer* (Lyman) (Ophiacanthidae), LAP, Recent, Japan. E: Ophiohelinae gen. nov. (Ophiacanthidae), parasol spine (GZG.INV.78779), Blake Nose. F: *Ophiotholia spathifer* (Lyman) (Ophiacanthidae), parasol spine, Recent, Japan. G: *Ophioleuce* sp. (Ophioleucinae), LAP (GZG.INV.78780), Blake Nose. H: *Ophioleuce seminudum* Koehler (Ophioleucinae), LAP, Recent, Pacific. I: *Ophiomusium* sp. (Ophiolepididae), LAP (a: external, b: internal) (GZG.INV.78781), Blake Nose. J: *Ophiomusium lymani* (Wyville-Thomson) (Ophiolepididae), LAP (a: external, b: internal), Recent, North Atlantic. K: Benthopectinidae gen. nov., ambulacral (GZG.INV.78782), Blake Nose. L: *Pectinaster filholi* Perrier (Benthopectinidae), ambulacral, Recent, Atlantic. M: Benthopectinidae gen. nov., adambulacral (GZG.INV.78783), Blake Nose. N: *Pectinaster filholi* Perrier (Benthopectinidae), adambulacral, Recent, North Atlantic. O: Laetmogonidae gen. nov., body wall ossicle (lower side) (GZG.INV.45613), Blake Nose. P: *Laetmogone olivacea* Théel (Laetmogonidae), body wall ossicle (lower side), Recent, North Atlantic. Q: *Hemisphaeranthos* sp. (Myriotrochidae), body wall ossicle (upper side) (GZG.INV.45634), Blake Nose. R: *Myriotrochus rinkii* Steenstrup (Myriotrochidae), body wall ossicle, Recent, North Atlantic. S: (?)*Myriotrochus* sp. (Myriotrochidae), radial calcareous ring element (inner side) (GZG.INV.45623), Blake Nose. T: *Myriotrochus rinkii* Steenstrup (Myriotrochidae), radial calcareous ring element (inner side), Recent, North Atlantic. Scale bars equal 100 µm.

## Results

We here present an echinoderm assemblage based on microfossils retrieved from sediments drilled by the Ocean Drilling Program (ODP) Leg 171B on the Blake Nose escarpment in the western tropical Atlantic, off Florida [13] (Fig. 1). The echinoderm remains described herein originate from the chalk succession recovered at ODP site 1049 underlying the black shales of Oceanic Anoxic Event (OAE) 1b [13] and dated as middle/late Aptian to earliest Albian (*Hedbergella trocoidea* to *Microhedbergella rischi* planktonic zones, ca 114 Ma) [14]–[15]. The geologic history of site 1049 implies deposition of the Aptian–Albian chalk succession at lower bathyal depths (at least 1500 m), whereas benthic foraminiferal assemblages indicate middle bathyal (800–1000 m) or greater depths [13], [16]. There was no input of periplatform debris at the time the chalk was deposited, as indicated by the lack of shallow-water foraminifera [13], and the echinoderm plates are thus interpreted to represent an autochthonous bathyal assemblage.

**Figure 3:**
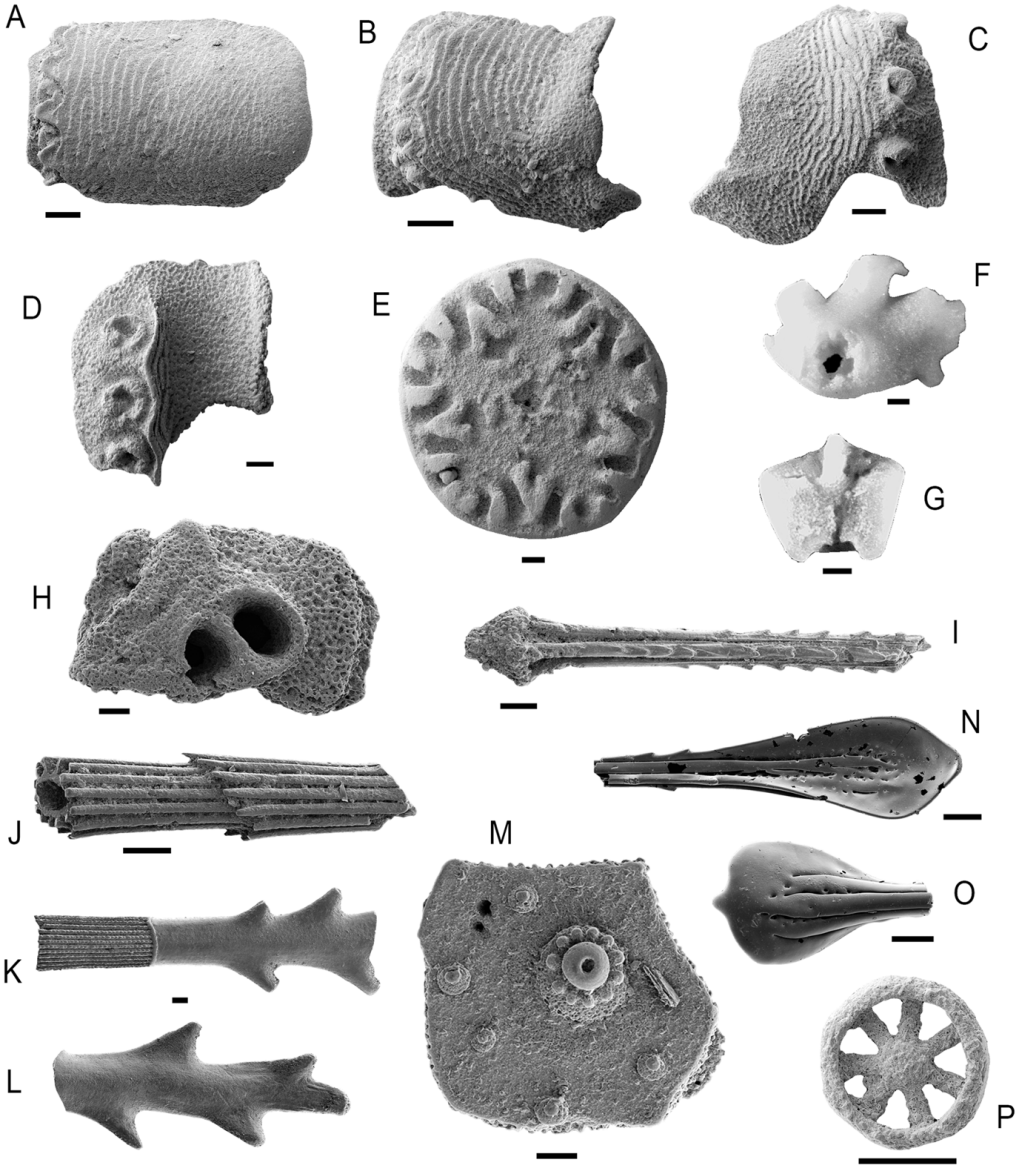
Additional diagnostic skeletal components of the echinoderm groups from the Aptian-earliest Albian (Early Cretaceous) of Blake Nose (ODP Site 1049). A: Ophiacanthidae gen. et sp. nov., lateral arm plate (GZG.INV.78784). B: *Ophiologimus* sp. nov. (Ophiacanthidae), lateral arm plate (GZG.INV.78785). C: *Ophiologimus* sp. nov. (Ophiacanthidae), lateral arm plate (GZG.INV.78786). D: *Ophiacantha* sp. nov. (Ophiacanthidae), lateral arm plate (GZG.INV.78787). E: *Balanocrinus* sp. (Isocrinidae), columnal (GZG.INV.78788). F: *Bathycrinus*? sp. (Bathycrinidae), holdfast (GZG.INV.78789). G: *Bathycrinus*? sp. (Bathycrinidae), second primibrachial (GZG.INV.78790). H: echinothurioid ambulacral plate (GZG.INV.78791). I: echinothurioid? spine (GZG.INV.78792). J: diadematoid spine (GZG.INV.78793). K-L: Histocidaridae gen. et sp. indet., adoral spine fragments (GZG.INV.78794–78795). M: holasteroid ambulacral plate (GZG.INV.78796). N: holasteroid spine fragment (GZG.INV.78797). O: holasteroid spine fragment (GZG.INV.78798). P: *Jumaraina* sp. (Chiridotidae) body wall ossicle (upper side) (GZG.INV.78799). Scale bars equal 100 µm.

### Taxonomic Composition

**Figure 4:**
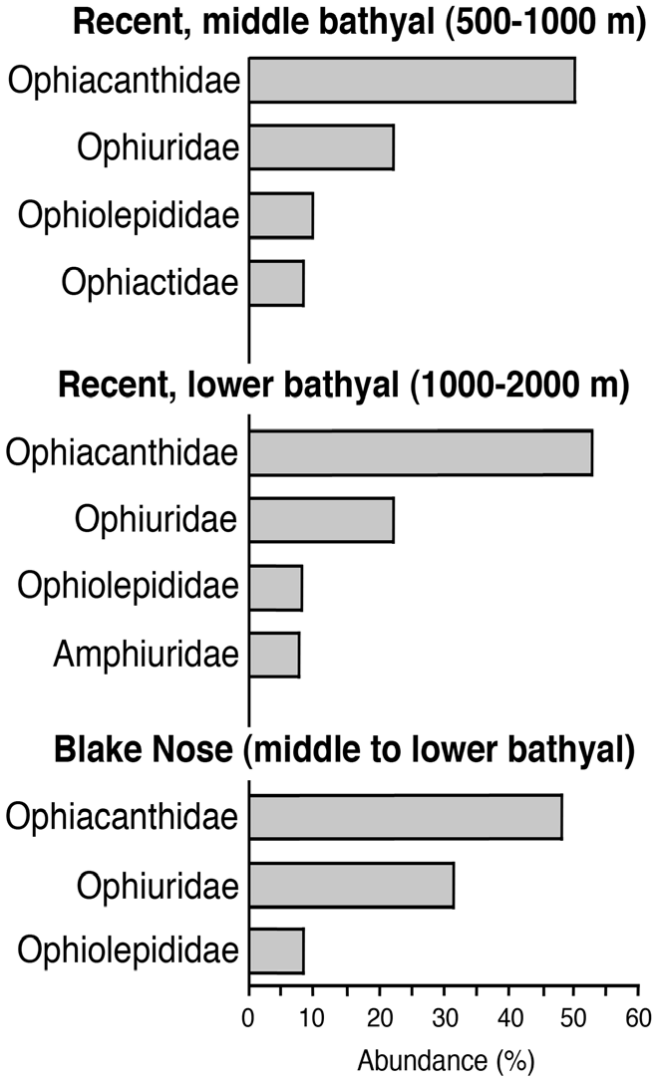
Relative abundances of the most common ophiuroid families in present-day middle and lower bathyal settings, in comparison with the middle to lower bathyal ophiuroid assemblages from the upper Aptian–lowermost Albian of Blake Nose (ODP site 1049). Relative family-level abundances of the Blake Nose ophiuroid assemblage were inferred from lateral arm plate counts, assuming that the number of lateral arm plates serves as an approximation for the number of individuals.

**Figure 5:**
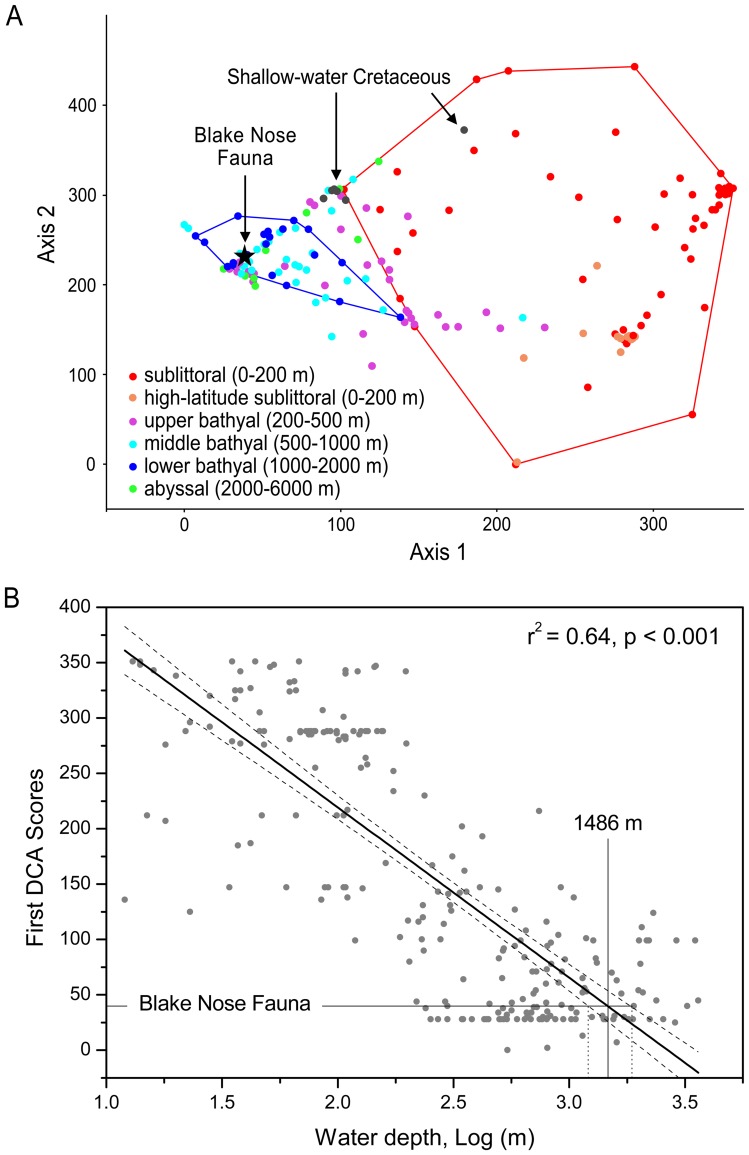
Quantitative assessment of the Blake Nose ophiuroid assemblage. A: Detrended Correspondence Analysis (DCA) of modern ophiuroid assemblages in comparison with the Blake Nose ophiuroid fauna and Cretaceous shelf assemblages. The Blake Nose assemblage plots within the modern lower bathyal communities, and strongly differs from modern shallow-water communities and Cretaceous shelf assemblages, challenging the possibility of repeated deep-sea recolonization from shelf depths in the Cretaceous. The analysis is based on the relative abundances of all 17 extant ophiuroid families minus the Ophiuridae, which are abundant at all depths (see Table 1 for abundance data). B: Linear correlation between DCA scores (axis 1) and LOG depth. The relationship is very strong (r  = −0.80, adjusted r-square = 0.64). The probability of such strong correlation occurring by chance is virtually zero (4.8899E−29). When the score of the Blake Nose fauna is projected onto this relationship, it would be assigned a depth of 1,486 m (1,218–1,864 m) (uncertainty based on 95% confidence interval for regression line). Remarkably, this is exactly within the range of paleodepth reconstructions for this site [13], [16]. This means the fauna is similar to present-day lower bathyal assemblages to such degree that even the faunal composition versus depth relationship appears to have remained the same.

The ophiuroid (brittle star) remains from the Blake Nose samples are composed mostly of the highly diverse, spine-bearing lateral arm plates [17]. Nearly half of these plates (48%) are assignable to the extant, predominantly deep-sea family Ophiacanthidae [18] on account of the ear-shaped spine articulations with a sigmoidal fold, the relatively thin aspect of the plates and a vertical series of perforations on the inner side (Figs 2A–F, 3A–D). Within this group, lateral arm plates with a conspicuous vertical striation including a minutely denticulate distal edge of the stripes, spine articulations sunken into the distal edge of the lateral arm plates, and well-developed connecting ridges between the spine articulations and the vertical stripes are assignable to the extant genus *Ophiolimna* (Fig. 2A–B). Lateral arm plates with a moderately to weakly developed, fine vertical striation and freestanding spine articulations on an elevated vertical ridge are commonly found in the extant genus *Ophiacantha* (Fig. 3D). Parasol-shaped arm spines similar to those found in the Blake Nose material are an exclusive feature of the modern deep-sea genera *Ophiohelus* and *Ophiotholia* (Fig. 2E–F). The co-occurrence of lateral arm plates with vertical and single (Fig. 2C–D) rather than oblique and multiple rows of spine articulations precludes assignment to *Ophiohelus* [19]. Most of the remaining lateral arm plates of the assemblage are assignable to the subfamily Ophioleucinae within the Ophiuridae (31%) (Fig. 2G–H), on account of the fragile plate architecture, the large tentacle notches, an outer surface ornamentation consisting of minute, scale-like granules and small rhombic spine articulations sunken into the distal edge of the plate, and to the extant deep-sea ophiolepidid genus *Ophiomusium* (8%) [18] (Fig. 2I–J). Lateral arm plates of the latter are typically rectangular in outline, with tentacle openings developed as within-plate perforations, and with a conspicuous large dorsal contact surface with the opposite lateral arm plate on the inner side. The composition at family level of the Blake Nose ophiuroid assemblage is closest to that of modern lower bathyal communities, and clearly differs from modern and Cretaceous shallow-water assemblages (Figs 4–5).

The asteroid (starfish) material from the Blake Nose samples (Fig. 2K–N) is dominated by the remains of a single taxon. The ambulacrals are hourglass shaped, with equally expanded triangular head and base, a benthopectinid synapomorphy. Furthermore, the ambulacral-adambulacral contact is highly modified [20] and unique to the Benthopectinidae, representatives of which are common elements of modern deep-sea asteroid assemblages [21].

The Blake Nose holothuroid (sea cucumber) assemblage consists of body wall wheels assignable to the Laetmogonidae on account of the presence of 6 to 15 spokes, a perforated hub with a central primary cross and a smooth rim (Fig. 2O–P). Body wall wheels presenting 10 to 20 spokes, a flat hub and a rim with small to medium-sized teeth are typical of the Myriotrochidae, a group which is furthermore represented in our material by calcareous ring elements the radial ones of which typically display a perforation for the passage of the radial nerve and long anterior prolongations (Fig. 2Q–T). A third type of wheels is characterized by the presence of 6 or rarely 7 to 8 spokes, a non-perforated hub which is complex at both sides, and a circular and finely denticulate rim, which unquestionably places them in the Chiridotidae (Fig. 3P). Modern myriotrochids and laetmogonids are typically bathyal groups, and, although not restricted to deep-sea settings, chiridotids are also commonly found in modern bathyal habitats [22].

The echinoid (sea urchin) assemblage studied herein includes spines with a smooth surface, a long collar, dorso-ventral flattening and ornamentation with coarse lateral thorns (Fig. 3K–L), a combination of characters known exclusively from the oral secondary spines of the extant family Histocidaridae. Strongly reduced ambulacral plates bearing a single large pore pair and lateral flanges bevelling under neighbor plates are assignable to the echinothurioids (Fig. 3H). Co-occurring secondary spines show morphologies consistent with those of extant echinothurioid secondary spines (Fig. 3I). Fragments of hollow, strongly verticillate spines were found in several samples, and clearly belong to a diadematoid echinoid (Fig. 3J), although attribution to any particular taxon within this large group is problematic. Almost all samples yield fragments and spines of thin-shelled atelostomate echinoids (Fig. 3M–O). Based on plate shape, ambulacral pore shape and position, as well as tuberculation, which consists of widely scattered primary tubercles with few distinctly smaller granules interspersed, an attribution to late stem-group holasteroids (tithoniids) [23] or early crown-group members seems likely. Although histocidarids, echinothurioids, diadematoids and holasteroids are typical components of modern deep-sea echinoid assemblages [6], the Blake Nose echinoid material fails to provide sufficient diagnostic elements for a detailed faunal analysis and comparison with modern equivalents. As predicted by phylogenetic analyses [24] our results indicate that echinothurioids and histocidarids evolved earlier than previously known, predating the oldest shallow-water occurrence of these groups. This may suggest that much of their evolution took place in deep-water settings.

The crinoid (sea lily) material from the Blake Nose assemblages includes columnals assignable to the isocrinid genus *Balanocrinus* on account of the short and uniform radiating crenulae along the margin, narrow radial ridges or ribbons of minute crenulae or granules, and large areolae (Fig. 3E). The assemblage furthermore comprises thin primibrachials 2 and a holdfast that are strongly reminiscent of the extant bourgueticrinid *Bathycrinus* (Fig. 3F–G). Modern isocrinids are found almost exclusively in bathyal settings, while *Bathycrinus*, and the modern bourgueticrinids in general, are among the deepest-dwelling of all extant crinoids, occurring from lower bathyal to hadal depths [25].

**Figure 6:**
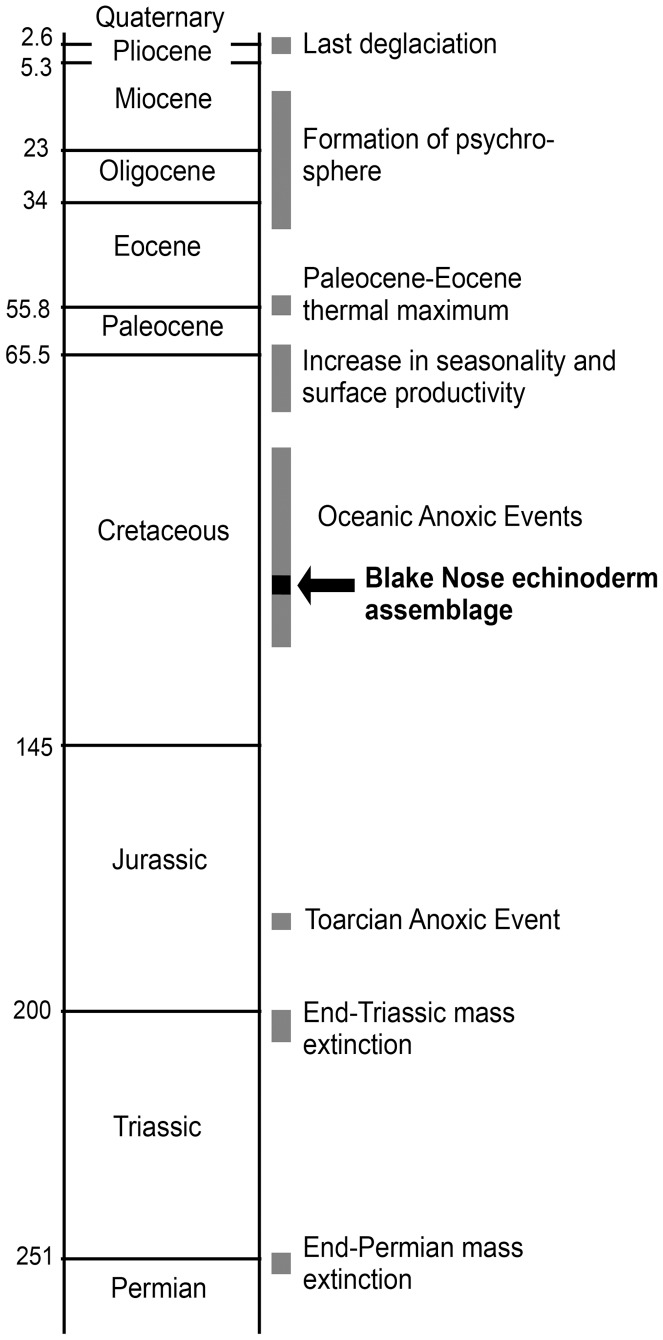
Position of the Blake Nose deep-sea echinoderm assemblage in the context of the events assumed to have triggered major reorganizations of the deep-sea fauna [**4]**–[6].

## Discussion

### Implications for the Origin of the Modern Deep-sea Fauna

The discovery of a middle to lower bathyal echinoderm community of modern composition (Figs 4–5) of Early Cretaceous age thus implies that a significant part of the modern deep-sea fauna is much older than currently assumed. As ongoing invasion of deep habitats evidently precludes the determination of a single point of origin for the entire modern deep-sea fauna [26], it seems more appropriate to attempt to approximate the last point of major deep-sea faunal reorganization.

**Table 1:** Ophiuroid lateral arm plate counts of the Blake Nose samples.

Section	Depth below surface (m)	Ophiacanthidae	Ophiuridae	Ophiolepididae	indeterminate
1049C 12×4 141–142	145,21	1	0	0	0
1049C 12×4 144–145	145,24	0	0	0	2
1049C 12×4 145–146	145,25	1	0	0	0
1049C 12×4 146–147	145,26	1	0	0	1
1049C 12×4 147–148	145,27	0	1	0	0
1049C 12×4 148–149	145,28	1	5	0	7
1049C 12×4 149–150	145,29	14	6	3	5
104912×5 0–1	145,30	23	17	1	6
104912×5 1–2	145,31	32	17	3	4
104912×5 3–4	145,33	5	4	0	1
104912×5 4–5	145,34	2	6	3	0
104912×5 5–6	145,35	5	2	3	1
104912×5 6–7	145,36	4	2	0	1
104912×5 7–8	145,37	4	7	0	2
104912×5 8–9	145,38	19	11	6	3
104912×5 9–10	145,39	30	12	1	4
104912×5 10–11	145,40	29	7	1	1
104912×5 11–12	145,41	15	6	0	7
104912×5 12–13	145,42	20	6	1	1
104912×5 13–14	145,43	8	16	1	2
104912×5 14–15	145,44	7	19	3	3
104912×5 15–16	145,45	9	14	0	1
104912×5 16–17	145,46	3	11	0	4
104912×5 17–18	145,47	5	7	0	0
104912×5 18–19	145,48	16	6	0	7
104912×5 19–20	145,49	27	24	5	2
104912×5 20–21	145,50	4	12	1	7
104912×5 21–22	145,51	14	15	1	10
104912×5 22–23	145,52	6	5	4	8
104912×5 23–24	145,53	8	13	4	12
104912×5 24–25	145,54	5	7	11	4
104912×5 25–26	145,55	3	13	4	6
104912×5 26–27	145,56	1	10	0	13
104912×5 27–28	145,57	3	12	0	5
104912×5 28–29	145,58	3	10	0	3
104912×5 29–30	145,59	3	2	0	1
104912×5 39–40	145,69	22	23	3	9
104912×5 59–60	145,89	33	40	7	2
104912×5 99–100	146,29	7	4	5	2
104912×5 139–140	146,69	18	6	1	1
1049C 12×6 0–1	146,80	4	1	0	3
1049C 12×6 40–41	147,20	11	5	0	7
1049C 12×6 60–61	147,40	29	0	0	4
1049C 12×6 70–71	147,50	8	0	6	1
1049C 12×6 100–101	147,80	24	0	2	5
1049C 12×6 120–121	148,00	6	0	3	3
1049C 13×1 0–1	148,90	10	0	0	2
1049C 13×1 30–31	149,20	9	3	0	5
1049C 13×1 60–61	149,50	1	0	0	3
1049C 13×1 130–131	150,20	107	8	13	4
1049C 13×1 140–141	150,30	29	2	0	6
1049C 13×1 40–41	150,80	45	37	3	5
1049C 13×1 50–51	150,90	12	5	12	2
1049C 13×1 60–61	151,00	9	5	1	6
1049C 13×1 80–81	151,20	41	11	1	1
1049C 13×1 100–101	151,40	14	6	3	1
1049C 13×1 130–131	151,70	3	0	0	0
1049C 13×1 149–150	151,89	9	12	7	7
1049A 20×3 80–81	157,30	0	1	0	0
1049A 20×4 60–61	158,60	7	2	1	4
1049A 20×4 70–71	158,70	5	5	2	2
1049A 20×4 100–101	159,00	63	54	22	1
1049A 20×4 110–111	159,10	13	9	3	11
1049A 20×4 140–141	159,40	2	3	1	3
1049A 20×4 150–151	159,50	33	19	8	3
1049A 20×4 10–11	159,70	68	57	8	8
1049A 20×4 30–31	159,90	3	6	2	2
1049A 20×4 50–51	160,10	3	5	0	0
Total	979	634	170	247	

Samples barren of ophiuroid remains were omitted; each sample represented 1 cm^3^.

The Blake Nose assemblage postdates the major OAE 1a (Aptian, 124 Ma), but predates the minor dysoxic events of Albian age (OAE 1b–d; 112–100 Ma), and the extensive and widespread OAE 2 (late Cenomanian, 93 Ma) that was associated with photic zone anoxia in the Atlantic [27]. The Paleocene–Eocene thermal maximum (PETM; 60 Ma) [28] and the mid-Cenozoic deep-water cooling events (34 Ma) [29] are much later (Fig. 6). We therefore suggest that all these events were less important in controlling deep-sea biodiversity than previously assumed [4]–[5], although, strictly speaking, our observation can be spatially extrapolated for the North Atlantic at most. Then again, in spite of extensive sampling especially in Upper Cretaceous shelf deposits, at least four of the echinoderm groups recorded from the Blake Nose section have no known post-Aptian fossil record at shelf depths. These are parasol-spined and ophioleucinid ophiuroids, benthopectinid asteroids (the Maastrichtian specimens described in references [11] and [30] are of uncertain and/or non-benthopectinid affinities), and laetmogonid holothuroids. This is a significant piece of evidence since it effectively precludes the possibility that deep-sea recolonization from shallow habitats following episodic extinction occurred in echinoderms. Rather, the evidence points to an early Mesozoic or older colonization of deep-sea habitats by the modern fauna.

It has been suggested that the Middle Jurassic fossil Lagerstätte of La Voulte, France, was deposited in a bathyal setting [31]. This paleodepth estimate is entirely based on the presence of hexactinellid sponges and stalked crinoids, modern representatives of which are largely restricted to the deep sea. There are, however, well-documented shallow-water fossil occurrences of these groups, suggesting that water depth is not the main factor controlling their distribution [12], [32]. Therefore, in the absence of a robust paleodepth reconstruction, the La Voulte fauna does not contribute significantly to a discussion of the geologic history of the modern deep-sea fauna.

Deep-sea vent and seep communities have been considered relatively resistant to extinction events [33]. The Blake Nose assemblage corroborates previous assumptions that this resistance, rather than a result of the independent food source of vent communities, is a phenomenon of deep-sea communities in general, despite the bentho-pelagic coupling through food delivery that was previously assumed to control deep-sea biodiversity [34]. It can be speculated that wide biogeographic distribution and great dispersal potential, commonly observed in modern deep-sea organisms [1], [26], might have played an important role in buffering the deep-water fauna against major extinction events. Whatever the causes, our data clearly show that the factors controlling biodiversity and the resilience of ecosystems to oceanographic changes differ significantly between deep-sea and shallow-marine settings.

The origin of much of the modern deep-sea fauna must be sought in sediments older than late Aptian. The two major Mesozoic oceanic anoxic events preceding the Blake Nose assemblage (Toarcian OAE and early Aptian OAE 1a) were unlikely to have been more severe than the end-Cenomanian OAE 2 [27], post-dating our assemblage. It thus seems likely that they had a similarly negligible effect on deep-sea biodiversity. We thus speculate that the end-Permian mass extinction, accompanied by a long-term, worldwide deep-sea anoxia [35] and, probably to a lesser extent, the end-Triassic extinction and productivity collapse [36] were potential triggers for the last major reorganization of deep-sea communities and thus the origin of a significant part of the modern fauna.

**Table 2:** Ophiuroid lateral arm plate counts of Cretaceous shallow-water assemblages.

Locality	Formation	Age	Ophiacanthidae	Ophiuridae	Ophiolepididae
Folkestone (UK)	Gault Clay, level 2	Albian	11	0	72
Folkestone (UK)	Gault clay, level 6	Albian	0	0	206
Mosqueruela (E)	Mosqueruela Formation	Aptian	17	9	535
Saginaw, Texas (USA)	upper Duck Creek Formation	Albian	0	0	100
Waco, Texas (USA)	Del Rio Formation	Cenomanian	1	0	58

## Methods

### Sample Selection and Treatment

Only few pre-Cenomanian *in situ* deep-sea sediments meet the requirements of bathyal paleodepth and good preservation of calcareous microfossils necessary to investigate deep-sea communities preceding the majority of the Cretaceous OAEs, in particular OAE 2. The most promising among them is ODP site 1049. A total of 74 samples, each representing 1 cm^3^ of sediment, from intervals 171B–1049C−12×4, 141 cm to 13×2, 81 cm, and 171B–1049A−20×3, 0 cm to 20×5, 51 cm, were examined. Sample treatment included mechanical disintegration in de-ionized water using an orbital shaker, and washing over a 0.063 mm screen. Echinoderm remains were picked from the >125 µm fraction, and, when appropriate, the >63 µm fraction. The material described herein was deposited in the micropaleontological collection at the University of Tübingen for the residues, and the Geoscience Museum at the University of Göttingen (GZG) for the echinoderm remains. Extraction of skeletal plates from Recent specimens for comparison was performed using household bleach for maceration.

### Quantitative Analysis

In order to compare the Blake Nose ophiuroid assemblage with modern brittle star communities in terms of abundance patterns, family-level specimen counts were compiled from various references covering all major ocean basins and depths ranging from shallow sublittoral to abyssal [37]–[44]. Abundance data for the Blake Nose assemblage were inferred from lateral arm plate counts (see Table 1), in which only unquestionably identifiable plates and fragments representing more than half a lateral arm plate were recorded. All other lateral arm plates and smaller fragments were counted as indeterminate and not included in the dataset. In addition, data from Cretaceous shallow-water ophiuroid communities were included in the analysis in order to test for possible similarities in family-level composition and abundance patterns with the bathyal Blake Nose assemblage. These were based on published Cretaceous assemblages [45]–[46], and on lateral arm plate counts of previously unpublished assemblages from the Aptian Mosqueruela Formation [47] of Mosqueruela (Spain), the middle Albian Gault Clay levels 2 and 6 [48] of Folkestone (United Kingdom), the upper Albian Duck Creek Formation of Saginaw, Texas (USA), and the Cenomanian Del Rio Formation [49] of Waco, Texas (USA) (See Table 2 for corresponding lateral arm plate counts). After removal of the Ophiuridae, ophiuroid assemblages with fewer than 10 individuals were deleted from the analysis to avoid overrating of rare components. In order to compare the above-mentioned assemblages, Detrended Correspondence Analysis (DCA) was performed using the PAST software [50].
